# Characterization Method for 3D Substructure of Nuclear Cell Based on Orthogonal Phase Images

**DOI:** 10.1155/2015/917640

**Published:** 2015-08-18

**Authors:** Ying Ji, Minjie Liang, Tingting Hua, Yuanyuan Xu, Zhiduo Xin, Yawei Wang

**Affiliations:** ^1^Faculty of Science, Jiangsu University, Zhenjiang 212013, China; ^2^School of Mechanical Engineering, Jiangsu University, Zhenjiang 212013, China

## Abstract

A set of optical models associated with blood cells are introduced in this paper. All of these models are made up of different parts possessing symmetries. The wrapped phase images as well as the unwrapped ones from two orthogonal directions related to some of these models are obtained by simulation technique. Because the phase mutation occurs on the boundary between nucleus and cytoplasm as well as on the boundary between cytoplasm and environment medium, the equation of inflexion curve is introduced to describe the size, morphology, and substructure of the nuclear cell based on the analysis of the phase features of the model. Furthermore, a mononuclear cell model is discussed as an example to verify this method. The simulation result shows that characterization with inflexion curve based on orthogonal phase images could describe the substructure of the cells availably, which may provide a new way to identify the typical biological cells quickly without scanning.

## 1. Introduction

The characteristics of biological cells usually contain the size, shape, and structure of the biological substances in the cells. These parameters of the cells are important for many researches in biology and life science since they are closely related to various functions and dynamics of the cells [1]. However, biological cells are mostly transparent objects, which make conventional intensity-based light microscopy techniques fail to image them owing to lack of the adequate contrast. Exogenous label contrast agents, such as fluorescence dyes, can solve the contrast problem, but they may make the living cells phototoxic and influence the cellular behavior [2]. Considering that optical phase delay may occur when light traverses through cells, phase microscopy, especially quantitative phase microscopy (QPM), as a noninvasive and nondestructive tool for imaging phase objects, plays an important role in the structure analysis, identification, and dynamic behavior analysis of the biological cells [3].

With the development of digital holography, the research on QPM technology in both cases of single-point and full-field phase imaging grows rapidly [4–10]. For the case of single-point, three-dimensional (3D) structure image of heterogeneous cells could be obtained, but the process is time-consuming. Thus it may not be effectively utilized for studying some dynamic imaging processes. As for the whole-field case, great satisfaction could be got from the data collected velocity as well as the spatial resolution of the image. However, many biological cells have substructural characteristics, such as five subtypes of white blood cells. Due to the coupling of the refractive index and the optical distance, only axial thickness of the cell can be obtained with the known refractive index. How to describe the 3D substructure of the cells in detail is still an open problem [11].

In this paper, typical optical models corresponding to blood cells are built for the demand of recognition of the subclasses of blood cells. Orthogonal phase images are applied to get the 3D substructure of the cell. The wrapped and unwrapped phase distribution maps are obtained from interference imaging. The characterization method for the morphology as well as the location of the cell nucleus is proposed by introducing inflexion-curve equation, which may have some reference for characterization of substructure features in quantitative phase imaging in the case of whole-field.

## 2. Cell Models of Nuclear Type and the Related Phase Distributions

**Figure 1:**
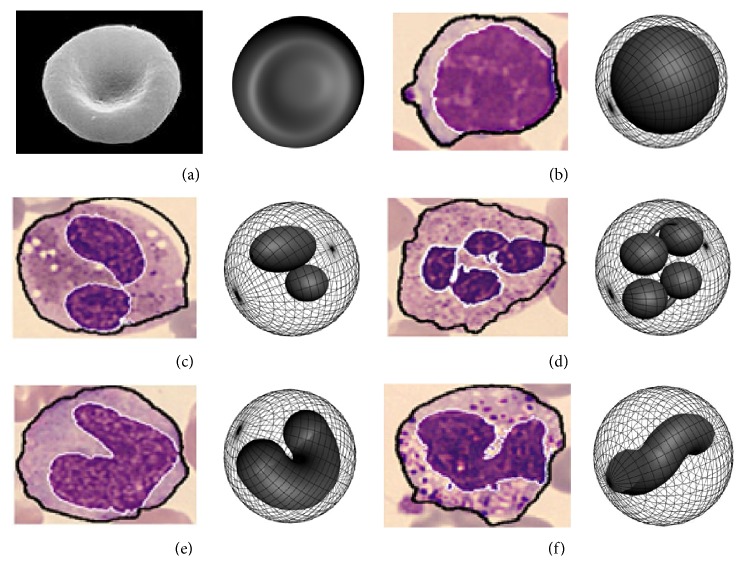
Morphological structure of typical blood cells and the associated 3D models. (a) RBC, (b) lymphocyte, (c) eosinophil, (d) neutrophil, (e) monocyte, and (f) basophil.

In clinical medicine, the blood test result, including the quantity and the morphology of which, is an important reference basis for the diagnosis and treatment of many diseases. Red blood cell (RBC) is a major kind of blood cell, playing an important role in the human body. Besides, there are white blood cells (WBC), which could be divided into five subtypes, that is, lymphocyte, eosinophil, neutrophil, basophil, and monocyte. The models of RBC and WBC have been built according to their optical characteristic and morphology (see Figure 1), in which the left one of each set is the morphological structure of the above typical blood cell which is quoted from [12]. Based on the characteristic information in respect of morphology, physiology, and physics, the associated 3D optical models of these cells have been built by VirtualLab simulation, which are demonstrated as the right ones of the sets, respectively [13]. To be specific, the model of RBC shows the shape of biconcave disc with a diameter of 7.7 *μ*m (seen in Figure 1(a)), whose thickness of the center section and edge section is 1.0 *μ*m and 2.0 *μ*m, respectively. The model of lymphocyte is made up of eccentric double spheres; that is, the external one with a diameter of 12.0 *μ*m denotes the membrane, while the internal one indicates the nucleus (shown in Figure 1(b)).  Figure 1(c) describes eosinophil. It looks like a sphere with two spheroids internally. The external sphere has a diameter of 12.0 *μ*m, which denotes the cell body. The model of neutrophil is demonstrated as a sphere with four spheroids internally (shown in Figure 1(d)). The diameter of the external sphere is 12.0 *μ*m and the internal four spheroids are separated into two pairs, which are connected to each other in one pair. The model of monocyte is described as a sphere with a U-shaped part internally. The sphere has a diameter of 12.0 *μ*m which denotes the membrane and the U-shaped part denotes the nucleus (shown in Figure 1(e)). Similar to the former case, an S-shaped part is put into a sphere to describe the basophil (seen in Figure 1(f)). It is worthy to note that each part of all models shown in Figure 1, that is, the nucleus and the cell membrane, is constructed by symmetrical geometries, which could be obtained by rotating the related outlines.

As for VirtualLab software as well as VirtualLab simulation, they are based on Maxwell electromagnetic field theory with combinations of the finite element method and image processing technology. The unified modeling platform is employed to ensure the complete compatibility of the lighting optical source and optical propagation properties. The reliability of this simulation platform has been proved by [14].

**Figure 2:**
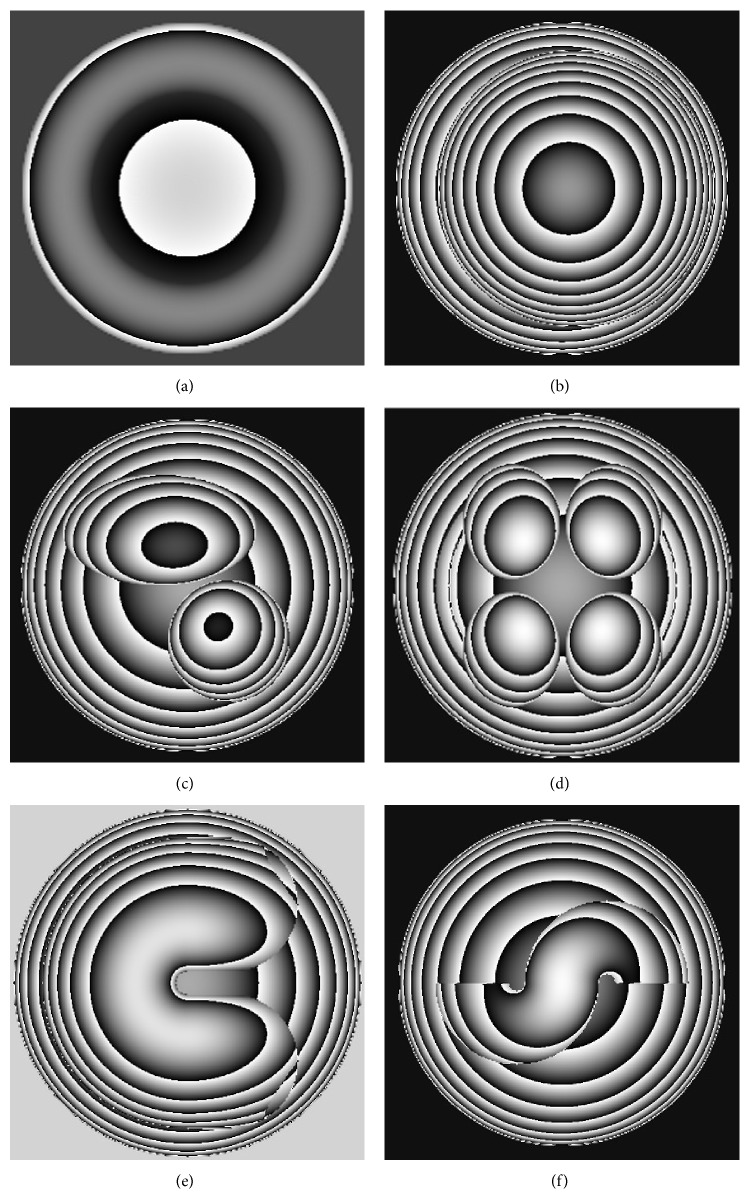
The wrapped phase maps corresponding to the models shown in Figure 1. Remark: the sequence number of each map in Figure 2 matches that in Figure 1 and it is also consistent in the following figures presented in this paper.

Based on the theory of phase detection, VirtualLab simulation is applied to get the associated phase images. The wrapped phase maps of the above six models are shown in Figure 2 [13]. In interferometry, phase distribution of the object is the integral of the optical path in longitudinal direction. Despite this, different phase images could be observed due to the different substructures of the models, respectively. This phenomenon suggests the feasibility to some extent that the substructure of the cell could be deconstructed from the information of the phase distribution.

## 3. Method to Reconstruct the 3D Substructure of the Cell

The inflexion curve is such a boundary shared by two contiguous areas on the surface, in which concave-convex directions are opposite to each other. It is considered to be a useful method for partition a surface into different concave-convex areas. The main idea of reconstruction is to record two orthogonal phase images of the object and then reconstruct the surfaces of the biological cells by means of geometric rotation related to the inflexion curves of the phase functions.

In phase image, the value of the phase has a mutation on the boundary between the nucleus and cytoplasm as well as on the boundary between cytoplasm and environment medium. It may be attributed to the mutations of cell morphology as well as the changes of the intracellular refractive indexes, which implies the existence of the nucleus. It is worth noting that the same mutations may be observed in both phase images from different directions only when cell nucleus exists. The outlines of the cell could be extracted from inflexion-curve equation. The information of structure is contained in the outlines of the cell. It is widely believed that the center point of one outline is the center of the 3D cell on the corresponding plane. Therefore, the center point of the cell could be determined by the comprehensive analysis of the two orthogonal outlines. Meanwhile, the equivalent volume, the ellipsoidal degree, and the phase center of nuclear could be defined to describe the size, morphology, and the central position of the nuclear. Finally, the space structure of the whole cell is merged by embedding the nucleus into the cytomembrane.

**Figure 3:**
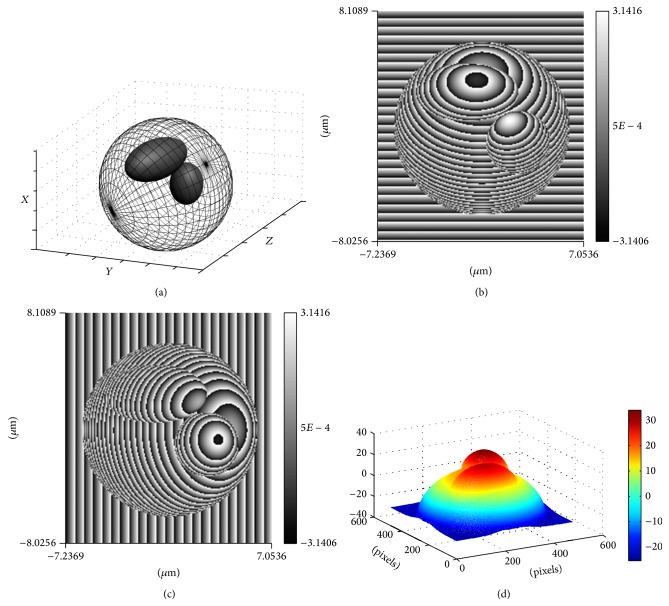
The phase maps for two-ellipsoid cell model of nuclear type. (a) The model of the cell of nuclear type. (b) The wrapped phase map on reference plane *x*-*y*. (c) The wrapped phase map on reference plane *x*-*z*. (d) The unwrapped phase map related to (b).

Here, we take a two-ellipsoid model of nuclear type as an example to explain the reconstruction process. The model is shown in Figure 3(a). Two orthogonal wrapped quantitative phase images of the specimen are presented as Figures 3(b) and 3(c). Combined with the associated unwrapped phase map of the cell shown in Figure 3(d), it is believed that two extreme values existed in the phase distribution.

The wrapped phase functions are defined as *φ*_1(*x*,*y*)_ and *φ*_2(*x*,*z*)_. Considering that the refractive index changes abruptly at the juncture of the cell nuclear and cytoplasm, which will lead the phase of incident wave to change relatively, one may search for the extremum of wrapped phase map to get the coordinate of the phase center on one reference plane. The second phase center on the other plane could be obtained by the same approach. In addition, the position of the phase center could be confirmed by geometry. That is, the center point of the closed phase contour in the projection plane of the wrapped phase map is considered to be the associated projection point of phase center. Thus, the phase center could be determined by detecting the two orthogonal wrapped phase maps. On the basis of the above analysis, the values of *x*-coordinate in different phase plane could be the same only if there exists cell nuclear because of the randomness of incident angle. Therefore, the coordinate values of the phase center could be obtained by means of this method.

In order to determine the equivalent volume and shape of the cell nuclear, the unwrapped phase functions *φ*_1*j*(*x*,*y*)_ and *φ*_2*j*(*x*,*z*)_ are obtained by phase unwrapping algorithm. The phase mutation may result in the concave-convex change of mathematical surface. Thus, demarcation line between cell nuclear and cytoplasm could be obtained by seeking the inflexion-curve equation of the phase function. For example, in order to get the inflexion curve on a projection plane, that is, plane *x*-*y*, one could get inflexion-curve equation *f*_1(*x*,*y*)_ = 0 with the definition of inflexion curve which is described as follows:

(1) $$\begin{array}{c} f_{1\left(x,y\right)}=\varphi_{1}^{\prime \prime }=\frac{\partial^{2}\varphi_{1j}}{\partial x^{2}}\frac{y_{x}^{\prime 2}}{1+y_{x}^{\prime 2}}-2\frac{\partial^{2}\varphi_{1j}}{\partial x\partial y}\frac{y_{x}^{\prime }}{1+y_{x}^{\prime }}+\frac{\partial^{2}\varphi_{1j}}{\partial y^{2}}\frac{1}{1+y_{x}^{\prime 2}}=0. \end{array}$$

Similarly, the inflexion-curve equation on plane *x*-*z*, that is, *f*_2(*x*,*z*)_ = 0, can be determined as well.

After the outlines of the substructure on different projection planes are extracted, the surface of the nuclear could be reconstructed by rotating an outline 180° around the perpendicular one. The equivalent volume and the ellipsoidal degree of cell nuclear could be obtained as well.

As for the structure of the whole cell, it could be determined by analyzing the phase function of the outside surface of the cell. The refractive indexes of the medium out of the cell, cytoplasm, and the nuclear are set at *n*_*m*_, *n*_1_, and *n*_2_, respectively, which are taken as known quantities. *h*_0_, *h*_1_, and *h*_2_ denote the axial thickness of the medium and outside surface of the cell and the nuclear. The unwrapped phase function of the whole cell along *z*-axis could be expressed as *φ*_1*j*(*x*,*y*)_ = 2*π*[(*n*_1_ − *n*_*m*_)*h*_1_ + (*n*_2_ − *n*_1_)*h*_2_]/*λ*. In view of the fact that subsurface could be embedded into the outside surface according to the position of the nuclear center determined above, the phase function of the outside surface could be written as *φ*_*j*(*x*,*y*)_ = *φ*_1*j*(*x*,*y*)_−2*π*[(*n*_2_ − *n*_1_)*h*_2(*x*,*y*)_]/*λ*, where *h*_2(*x*,*y*)_ means the axial thickness of the nuclear otained from the previous step. Thus, the thickness distribution of the outside surface could be obtained and the 3D substructure of the whole nucleated cell may be achieved combined with the analysis of the the nuclear subsurface as well.

## 4. Simulation Verification

**Figure 4:**
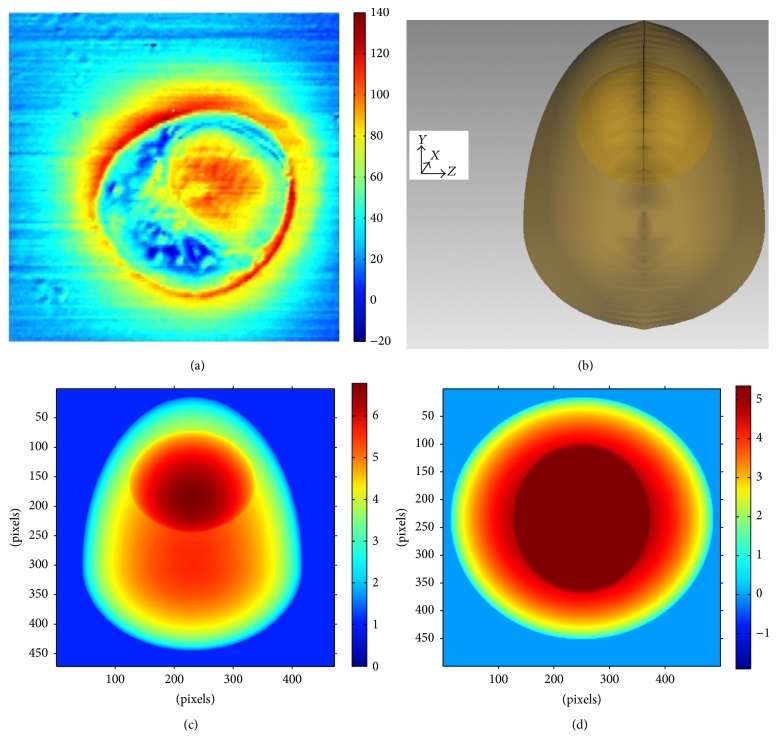
The mononuclear cell. (a)The real phase image from Kert Edward's work. (b) The model of a mononuclear cell. (c) The unwrapped phase map on reference plane *y*-*z*. (d) The unwrapped phase map on reference plane *x*-*z*.

In order to verify the effectiveness of this characterization method, a kind of common WBC, that is, monocyte, has been taken as a phase object to display the reconstruction process of the nucleus by simulation. The real phase image as well as the optical model is shown in Figures 4(a) and 4(b) [11, 12].

According to Kert Edward's work, the monocyte model is composed of two parts, that is, cytomembrane and nucleus. The cytomembrane consists of two half-ellipsoids with different semiaxis lengths. The nucleus of the cell is an ellipsoid with the semiaxis lengths along *x*, *y*, and  *z* directions set at 3.40 *μ*m, 3.70 *μ*m, and 3.00 *μ*m. The values of refractive index of the cytoplasm and the nucleus are set as 1.37 and 1.39, respectively. The incident wave is assumed to be a plane wave with the wavelength 488 nm in simulation experiment of phase imaging. The reference wave has the same wavelength as the incident wave with the initial phase of zero and the refractive index of environment medium is 1.003. The unwrapped phase maps of the model from two vertical directions are depicted in Figures 4(c) and 4(d).

**Figure 5:**
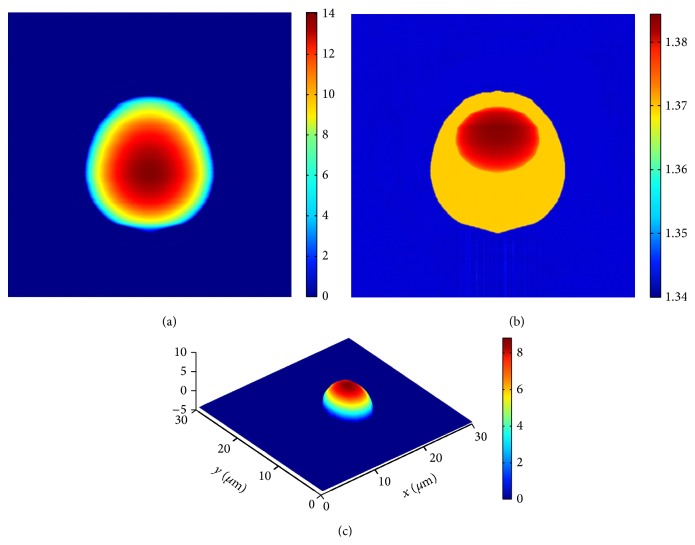
Simulation results of a mononuclear cell. (a) The axial physical thickness distribution of the whole cell. (b) The axial refractive index distribution of the whole cell. (c) The 3D substructure of the nucleus.

By comparing with the phase map obtained from real experiment [12] (see Figure 4(a)), one may find that there is good consistency between simulation and experiment, which may indicate the availability of this model to some extent. The distribution of the physical thickness as well as the refractive index distribution of the whole cell along *y*-axis is demonstrated as Figures 5(a) and 5(b), respectively.

Considering that the information of the nucleus is very important to morphological identification of the whole cell, the physical structure and the related parameters of the nucleus are focused on in this paper. By means of extracting and rotating the associated inflexion curves, the 3D substructure of nucleus is described as Figure 5(c). The related results of numerical calculations are shown in Table 1. By comparing the associated values preset above, it could be found that the simulation results agree well with the original setting situation.

## 5. Conclusions

A geometrical substructure reconstruction method is presented in this paper. The models of blood cells, including red blood cell and five subtypes of white blood cell, have been introduced in this paper. The orthogonal quantitative phase images of part models of nuclear type are obtained by simulation technique based on holographic phase interference. Furthermore, inflexion-curve equations of the nuclear and the cytomembrane can be extracted, respectively. And the 3D substructure of a nucleus is obtained by rotating the associated outlines. The simulation results agree well with the practical case. This method could be applied to rapid characterization or identification of mass blood cells with symmetric substructural characteristics without scanning.

It should be noted that the method still has a few limitations since the symmetry of specimen is required. To solve this problem, more phase images could be employed to get more inflexion curves, so that the substructure of the cell could be obtained by combining different parts associated with different inflexion curves. Thus, the reconstruction result could be more exact. It is our next work to apply this method to more general biological sample.

## Figures and Tables

**Table 1:** The numerical result of reconstructed cell nucleus.

Direction	Coordinate value of the nucleus	Preset values of the coordinate	Semimajor axis of the nucleus	Preset values of the semimajor axis
*x*	0.01 *μ*m	0.00 *μ*m	3.36 *μ*m	3.40 *μ*m
*y*	0.003 *μ*m	0.00 *μ*m	3.79 *μ*m	3.70 *μ*m
*z*	3.03 *μ*m	3.00 *μ*m	2.96 *μ*m	3.00 *μ*m
